# A Simple Optical Model Well Explains Plasmonic-Nanoparticle-Enhanced Spectral Photocurrent in Optically Thin Solar Cells

**DOI:** 10.1186/s11671-016-1449-y

**Published:** 2016-05-03

**Authors:** Katsuaki Tanabe

**Affiliations:** Department of Chemical Engineering, Kyoto University, Nishikyo, Kyoto 615-8510 Japan

## Abstract

A simple optical model for photocurrent enhancement by plasmonic metal nanoparticles atop solar cells has been developed. Our model deals with the absorption, reflection, and scattering of incident sunlight as well as radiation efficiencies on metallic nanoparticles. Our calculation results satisfactorily reproduce a series of experimental spectral data for optically thin GaAs solar cells with Ag and Al nanoparticles of various dimensions, demonstrating the validity of our modeling approach. Our model is likely to be a powerful tool for investigations of surface plasmon-enhanced thin-film solar cells.

## Background

Solar cell structures have been suffering from the following trade-off related to the thickness of their active photovoltaic layers: thinner photovoltaic layers exhibit weaker light absorption while thicker layers exhibit stronger bulk carrier recombination. Both of these factors yield conversion loss of the incident sunlight energy to the solar cell electrical output. Therefore, the thickness of the active photovoltaic layer is usually optimized for maximizing the energy conversion efficiency by considering the above trade-off. Metal nanoparticles placed on the solar cell surface can enhance sunlight collection, owing to their large extinction cross-section near the surface plasmon resonance, which is dominated by scattering rather than by absorption for appropriately chosen particle sizes [1–12]. Thus, metal nanoparticles scatter the incident light into a wide range of angles and increase the optical path length in the absorber layer for enhancing overall photoabsorption. This effect can potentially allow to reduce the cell cost and weight by utilization of thinner absorber layers and can also yield efficiency enhancement associated with an increased carrier excitation level. We previously experimentally investigated the effect of arrays of subwavelength-sized metal particles on GaAs solar cell absorption and photocurrent [9]. Spectral response measurements for optically thin GaAs solar cells, in which the photovoltaic active layer is much thinner than the optical absorptive decay length, were performed with and without Ag and Al metal nanoparticles; short circuit current and efficiency enhancement were observed under the air mass 1.5 global solar spectrum for GaAs cells with metal nanoparticle arrays, relative to reference GaAs cells with no metal nanoparticles.

Research groups have been primarily using the laborious finite-difference time-domain calculations to analyze or design surface plasmon-enhanced solar cells. However, such monochromatic, three-dimensional time-domain calculations are time-consuming, typically requiring more than several tens of hours of calculation by relatively powerful computers, even for a single wavelength of incident sunlight. In the present work, we propose and demonstrate a simple numerical simulation scheme for obtaining photocurrent enhancement spectra of plasmonic solar cells, which enables obtaining instant results for the entire sunlight spectrum, for providing future directions for device improvement. We demonstrate that our computational scheme is quite simple yet satisfactorily reproduces the experimental results for the photocurrent enhancement in solar cells with metal nanoparticle surface decorations.

## Methods

**Fig. 1:**
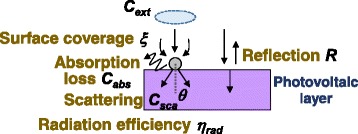
(Color online) cross-section schematic of the optical system modeled for metal-nanoparticle-enhanced solar cells

A simple optical model, representing metal nanoparticle surface plasmon resonances and multi-angle scattering, has been developed to reproduce and understand the spectral behavior of the experimental photocurrent enhancement and thus the role of metal nanoparticles in optically thin solar cells. We calculated the GaAs cell absorbance by considering scattering and absorption by the metal nanoparticles, by accounting for the nanoparticles’ surface coverage, reflectivity at the air/GaAs interface, angular dependence of scattered light, extinction efficiency factor (corresponding to the extinction cross-section of the nanoparticles normalized by the geometrical cross-section, and parameterizing the effect of the incident light on the nanoparticles), and radiation efficiency, which quantifies the relative prevalence of scattering over absorption for light that interacts with the nanoparticles. We calculated these factors for oblate spheroid nanoparticles in the quasistatic limit by using an effective medium approximation accounting for the influence of both air and GaAs. The calculation details follow. The detailed description of the structure and fabrication method of the experimental optically thin GaAs solar cells is given in [9]. For simplicity, we considered only the GaAs photovoltaic layer, neglecting the AlGaAs window layer. (Note that the refractive indices of GaAs and AlGaAs are similar.) The absorption fraction of the incident light in a GaAs layer of thickness *L* is

1 $$ {A}_0\left(\lambda \right)=1- \exp \left(-\alpha \left(\lambda \right)L\right), $$

according to the Beer-Lambert law for attenuation of light, where *α* is the absorption constant of GaAs and *λ* is the wavelength in a vacuum. In this work, we set *L* to 200 nm for the *p-n* diode photovoltaic active layer (a 50-nm-thick *p*-GaAs emitter + a 150-nm-thick *n*-GaAs base) as in [9]. The angular distribution of the light intensity scattered by subwavelength-sized particles in the quasistatic limit is

2 $$ {I}_{\mathrm{sca}}\propto \left(1+{ \cos}^2\theta \right){I}_0, $$

where the angle *θ* is measured between the forward and scattering directions [13]. Note that the quasistatic approximation used in this study is valid for the particles smaller than the wavelength of light, for which the phase retardation is negligible throughout the particle. The absorption fraction for the scattered light is

3 $$ {A}_{\theta}\left(\lambda \right)={\displaystyle {\int}_0^{\pi /2}\frac{1+{ \cos}^2\theta }{{\displaystyle {\int}_0^{\pi}\left(1+{ \cos}^2\theta \right)d\theta }}}\left\{1- \exp \left(-\alpha \left(\lambda \right)\frac{L}{ \cos \theta}\right)\right\}d\theta, $$

accounting for the optical path increase in the GaAs layer, from *L* into *L/cos*θ. Figure 1 shows a schematic of the optical system considered in this study. In this study, we did not account for the reflection of the scattered light at the metal/semiconductor interface but simply assumed that all the forward-scattered components directly enter the semiconductor layer. The phase relations among the transmitted and scattered field components were not accounted, either, for simplicity in our present model, while some interference may practically occur among the photons scattered by neighboring metal nanoparticles for the transverse spatial coherence length of the sunlight of several tens of micrometers [14, 15], significantly larger than the interparticle spacings. However, the effect of constructive and destructive interference will perhaps be eventually areally balanced and averaged not to matter in the result of total cell absorption. Interference may also occur between the components transmitted and scattered by metal particles [16] because the sunlight longitudinal coherence length is several hundreds of nanometers [15, 17, 18], comparable or larger than the thickness of the semiconductor photovoltaic layers, which in contrast may affect the cell absorption spectra. These issues indicate a room for improvement in our model. The total absorption fraction for the GaAs layer with nanoparticles on top is

4 $$ {A}_{\mathrm{tot}}\left(\lambda \right)=\varPhi \left(\lambda \right){\eta}_{\mathrm{rad}}\left(\lambda \right){A}_{\theta}\left(\lambda \right)+\left(1-\varPhi \left(\lambda \right)\right)\left(1-R\left(\lambda \right)\right){A}_0\left(\lambda \right), $$

where we define the light-coverage fraction *Φ*, which represents the areal fraction of the incident light interacting with the metal nanoparticles; this fraction is explained below. The parameter *η*_rad_ is the radiation efficiency, representing the ratio of the scattering cross-section to the extinction cross-section for the nanoparticles as defined in [2], and will also be explained below. The spectral wavelength-dependent reflectivity *R* at the air/GaAs interface was also accounted for as follows, because our GaAs solar cells had no anti-reflective coating or surface structure. Assuming normal incidence of light onto a GaAs layer, the wavelength-dependent Fresnel reflectivity at the air/GaAs interface is

5 $$ R\left(\lambda \right)\equiv \frac{I_r\left(\lambda \right)}{I_0\left(\lambda \right)}={\left|\frac{N_2\left(\lambda \right)-{N}_1\left(\lambda \right)}{N_2\left(\lambda \right)+{N}_1\left(\lambda \right)}\right|}^2, $$

where *N* is the complex refractive index of air or GaAs. *Φ* was calculated as follows, accounting for the geometrical overlap among the extinction cross-sections of the metal nanoparticles and by assuming a triangular lattice for the two-dimensional array of nanoparticles:

6 $$ \varPhi =\xi {Q}_{\mathrm{ext}},\kern0.5em \xi {Q}_{\mathrm{ext}}<\frac{\pi }{2\sqrt{3}} $$

7 $$ \varPhi =4\sqrt{3}\left[\frac{1}{2}\sqrt{\frac{\sqrt{3}}{2\pi}\xi {Q}_{\mathrm{ext}}} \sin \left\{ \arccos \left(\frac{1}{2}\sqrt{\frac{2\pi }{\sqrt{3}\xi {Q}_{\mathrm{ext}}}}\right)\right\}+\frac{\sqrt{3}}{2\pi}\xi {Q}_{\mathrm{ext}}\left\{\frac{\pi }{6}- \arccos \left(\frac{1}{2}\sqrt{\frac{2\pi }{\sqrt{3}\xi {Q}_{\mathrm{ext}}}}\right)\right\}\right],\kern0.5em \frac{\pi }{2\sqrt{3}}\le \xi {Q}_{\mathrm{ext}}<\frac{2\pi }{3\sqrt{3}}, $$

8 $$ \varPhi =1,\kern0.5em \xi {Q}_{\mathrm{ext}}\ge \frac{2\pi }{3\sqrt{3}}. $$

**Fig. 2:**
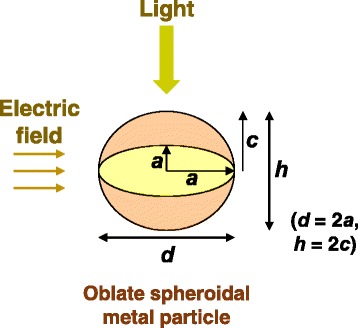
(Color online) Schematic of the geometry of the light-particle interaction system

The two conditional boundary points of *ξQ*_ext_ represent the fractions at which the extinction cross-sections start to overlap with one another and at which the extinction cross-sections had totally filled up the entire surface, respectively. *ξ* is the surface coverage fraction of the metal nanoparticles on the top surface of a solar cell, which is 0.4 and 0.3 for the particle diameters *d* of 60 and 150 nm, respectively, as determined from scanning electron microscope images of our experimental data in [9]. *Q*_ext_ is the extinction efficiency factor, which is the ratio of the extinction cross-section to the geometrical cross-section, as defined in [13]. *ξQ*_ext_ and *η*_rad_ were calculated as follows, based on the classical electromagnetic field theory in the quasistatic limit for oblate spheroidal metal particles with a minor axis parallel to the incident light corresponding to the height *h* of the experimental nanoparticles [13]. The absorption cross-section *C*_abs_ and the scattering cross-section *C*_sca_ of subwavelength-sized particles in response to incident light are

9 $$ {C}_{\mathrm{abs}}\left(\lambda \right)=k\mathrm{I}\mathrm{m}\left\{{\alpha}_1\left(\lambda \right)\right\}, $$

10 $$ {C}_{\mathrm{sca}}\left(\lambda \right)=\frac{k^4}{6\pi }{\left|{\alpha}_1\left(\lambda \right)\right|}^2, $$

where *k* and α_1_ are the wave number of light and the polarizability of the particle along the direction parallel to the electric field lines (i.e., vertical to the light propagation direction). Figure 2 shows the schematic of the geometry of the light-particle interaction system in this study. The polarizability of spheroidal particles is

11 $$ {\alpha}_j\left(\lambda \right)=4\pi abc\frac{\varepsilon_1\left(\lambda \right)-{\varepsilon}_m\left(\lambda \right)}{3{\varepsilon}_m\left(\lambda \right)+3{L}_j\left({\varepsilon}_1\left(\lambda \right)-{\varepsilon}_m\left(\lambda \right)\right)}, $$

where *a*, *b*, and *c* are the radii of the spheroid axes, *ε*_1,_ and *ε*_m_ are the complex dielectric functions of the particle and the surrounding medium, and *L*_*j*_ is the geometrical factor. The correspondence with the metal particles considered in this study is in that the experimental particle diameter and height are *d* = 2*a* and *h* = 2*c*, respectively, obtained by approximating the experimental particle shape as oblate spheroidal. *L*_*j*_ is calculated as

12 $$ {L}_j=\frac{abc}{2}{\displaystyle {\int}_0^{\infty}\frac{dq}{\left({l}^2+q\right)\sqrt{\left(q+{a}^2\right)\left(q+{b}^2\right)\left(q+{c}^2\right)}}}, $$

where *l* is *a*, *b*, or *c*, corresponding to *j* = 1, 2, 3, respectively. Note that, in general, 0 < *L*_*j*_ < 1, and *L*_1_ = *L*_2_ = *L*_3_ = 1/3 for spherical particles. The result of the computation based on Eq. 12 for oblate spheroids is

13 $$ {L}_1=\frac{\sqrt{1-{e}^2}}{2{e}^3}\left\{\frac{\pi }{2}- \arctan \left(\frac{\sqrt{1-{e}^2}}{e}\right)\right\}-\frac{1-{e}^2}{2{e}^2}, $$

where *e* is the eccentricity of the particle shape,

14 $$ e=\sqrt{1-\frac{c^2}{a^2}}. $$

We obtain *Q*_ext_ and *η*_rad_ by

15 $$ {Q}_{\mathrm{ext}}\left(\lambda \right)\equiv \frac{C_{\mathrm{ext}}\left(\lambda \right)}{G}=\frac{C_{\mathrm{abs}}\left(\lambda \right)+{C}_{\mathrm{sca}}\left(\lambda \right)}{\pi {a}^2}, $$

where *C*_ext_ is the extinction cross-section, corresponding to the sum of *C*_abs_ and *C*_sca_, and *G* is the geometric cross-section of the particle, which is *πa*^2^ in our case. We obtain *η*_rad_ as

16 $$ {\eta}_{\mathrm{rad}}\left(\lambda \right)\equiv \frac{C_{\mathrm{sca}}\left(\lambda \right)}{C_{\mathrm{ext}}\left(\lambda \right)}=\frac{C_{\mathrm{sca}}\left(\lambda \right)}{C_{\mathrm{sca}}\left(\lambda \right)+{C}_{\mathrm{abs}}\left(\lambda \right)}. $$

Various numerical schemes to take into account the effect of semiconductor substrates on the optical property of metal particles have been investigated [19–22]. In this study, we instead use a simple effective medium approximation for the complex dielectric function of the medium surrounding the metal nanoparticles:

17 $$ {\varepsilon}_{\mathrm{medium}}\left(\lambda \right)=\frac{2{\varepsilon}_{\mathrm{air}}\left(\lambda \right)+{\varepsilon}_{\mathrm{GaAs}}\left(\lambda \right)}{3}. $$

The above expression accounts for the influence of air and GaAs on the metal nanoparticles. The weight between the air and GaAs, 2:1, in Eq. 17 is the sole fitting parameter in our model calculations, consistently applied for all the series of data presented in this paper. The wavelength-dependent complex dielectric functions of metals and GaAs were obtained from [23]. For Al nanoparticles, in particular, *Q*_ext_ and *η*_rad_, were calculated for concentric Al-Al_2_O_3_ core-shell spheroidal particles with an Al_2_O_3_ shell thickness *t* of 4 nm, accounting for surface oxidation of Al nanoparticles in the ambient air [24, 25]. For concentric core-shell spheroidal nanoparticles, the polarizability along an axis is [13].

18 $$ {\alpha}_j=4\pi abc\frac{\left({\varepsilon}_2-{\varepsilon}_m\right)\left\{{\varepsilon}_2+\left({\varepsilon}_1-{\varepsilon}_2\right)\left({L}_j^{(1)}-f{L}_j^{(2)}\right)\right\}+f{\varepsilon}_2\left({\varepsilon}_1-{\varepsilon}_2\right)}{\left\{{\varepsilon}_2+\left({\varepsilon}_1-{\varepsilon}_2\right)\left({L}_j^{(1)}-f{L}_j^{(2)}\right)\right\}\left\{{\varepsilon}_m+\left({\varepsilon}_2-{\varepsilon}_m\right){L}_j^{(2)}\right\}+f{L}_j^{(2)}{\varepsilon}_2\left({\varepsilon}_1-{\varepsilon}_2\right)}, $$

where *ε*_1_, *ε*_2_, and *ε*_m_ are the complex dielectric functions of the core, shell, and the surrounding medium, respectively; *L*_*j*_^(1)^ and *L*_*j*_^(2)^ are the geometrical factors of the core and shell; and *f* is the volume ratio factor, which in our case is given by

19 $$ f\equiv \frac{\left(a-t\right)\left(b-t\right)\left(c-t\right)}{abc}=\frac{{\left(a-t\right)}^2\left(c-t\right)}{a^2c}. $$

Note that *a*, *b*, and *c* are the outer radii of the spheroid or the core radii. To compare with the experimental photocurrent enhancement data, we considered the ratio of the absorbance from Eq. 4 to the absorbance without nanoparticles. In this study, we assumed that the absorption enhancement in the photovoltaic active layer of a cell with metal nanoparticles relative to a reference cell without metal nanoparticles represents the photocurrent enhancement.

## Results and Discussion

**Fig. 3:**
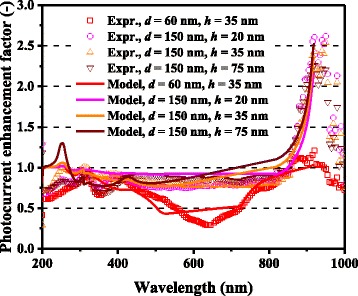
(Color online) Spectral photocurrent enhancement factors of optically thin GaAs solar cells with Ag nanoparticles

**Fig. 4:**
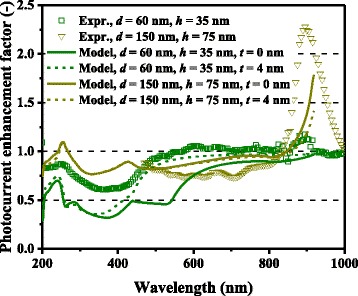
(Color online) Spectral photocurrent enhancement factors of optically thin GaAs solar cells with Al nanoparticles

**Fig. 5:**
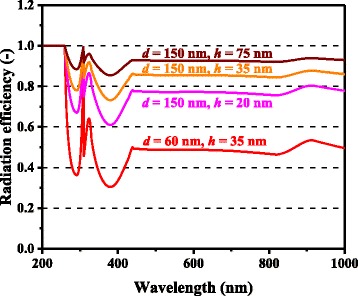
(Color online) Calculated optical radiation efficiencies of Ag nanoparticles of different dimensions

In Figs. 3 and 4, the computed photocurrent enhancement factors, defined as the ratio of the photocurrent between the cells with and without metal nanoparticles, are shown along with the experimental plasmonic-GaAs-cell data from [9]. The model calculation results are in a good qualitative agreement with the experimental results, including the peaks at ~300 and ~900 nm and the dips at ~600 nm for the 60-nm-diameter Ag nanoparticles and ~350 nm for the 60-nm-diameter Al nanoparticles. The observed photocurrent enhancement is thus attributed to the scattering effects of metal nanoparticles for light incident onto photovoltaic layers, which is captured by our model. The photocurrent enhancement factor is observed to be significantly higher for the cells with 150-nm-diameter Ag nanoparticles relative to those with 60-nm-diameter nanoparticles, for almost the entire spectral range. The dip in the photocurrent enhancement factor at ~600 nm for the cell with 60-nm-diameter Ag nanoparticles, presumably owing to the surface plasmon resonance of the Ag nanoparticles, is not observed for 150-nm-diameter nanoparticles. These results can be attributed to the considerably higher *η*_rad_ (~0.9) for the case of the 150-nm-diameter nanoparticles than that (~0.6) for the case of the 60-nm-diameter nanoparticles, suppressing the absorption loss in the Ag nanoparticles, as shown in Fig. 5. *η*_rad_ is an important factor, which represents how much incident light interacting with the nanoparticles is scattered rather than being absorbed, resulting in loss; thus, this parameter quantifies the cell performance. The scattering-to-absorption-rate ratio increases for larger nanoparticles in the quasistatic limit approximation that is valid for subwavelength-scale particles, while particles with sizes comparable to or larger than incident wavelengths are likely to suffer from electrodynamic damping, causing solar energy loss through particle heating [26–28]. It should be noted that surface plasmon resonance with relatively absorptive metal nanoparticles will provide rather negative effects particularly for shorter wavelength regions where the original bare cells had sufficient sunlight absorption in their semiconductor active layers. Plasmonic particles with high optical radiation efficiencies can, in other words, positively harness their supportive ability to enhance the effective absorption length in relatively thin photovoltaic layers at longer wavelengths. A degree of care is thus required for the manufacturers to properly measure which cases to install metal nanoparticles or not.

**Fig. 6:**
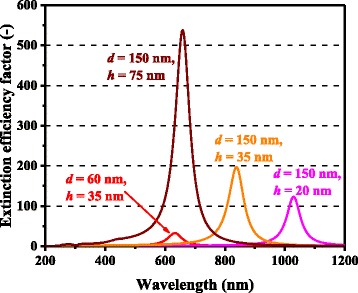
(Color online) Calculated extinction efficiency factors of Ag nanoparticles of different dimensions

**Fig. 7:**
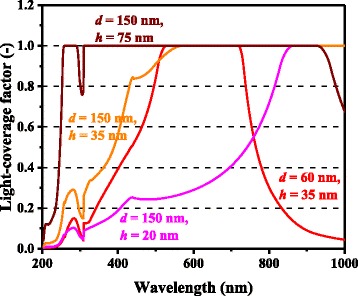
(Color online) Calculated light-coverage fractions of Ag nanoparticles of different dimensions

**Fig. 8:**
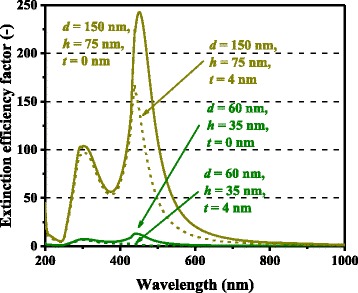
(Color online) Calculated extinction efficiency factors of Al nanoparticles of different dimensions

**Fig. 9:**
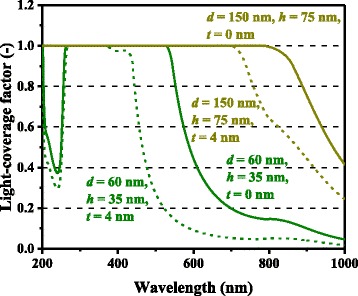
(Color online) Calculated light-coverage fractions of Al nanoparticles of different dimensions

**Fig. 10:**
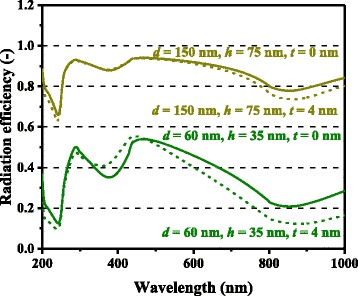
(Color online) Calculated optical radiation efficiencies of Al nanoparticles of different dimensions

For both Ag and Al, higher photocurrent enhancement at 900 nm for the 150-nm-diameter cases compared with the 60-nm-diameter cases is captured by the model and is caused mainly by higher *Q*_ext_ for larger metal nanoparticles. *Q*_ext_ quantifies how much light or electromagnetic field intensity is collected by a particle (similar to the treatment for antennae), relative to its geometrical size. Figure 6 shows the *Q*_ext_ spectra of Ag nanoparticles of various dimensions. The peaks in Fig. 6 represent the plasmon resonances for each nanoparticle type; the peak location is determined by the aspect ratio, *h/d*, of the nanoparticle shape. This result suggests that Ag nanoparticles collect a significant amount of incident light to interact with themselves, resulting either in scattering or absorption. Thus, very densely packed arrays of metal nanoparticles are not necessary but relatively disperse nanoparticle arrays can ensure sufficient collection of incoming light for scattering it into shallower angles to fully harvest the absorption enhancement, as indicated in Fig. 7, which shows the *Φ* spectra for the experimental data of cells with Ag nanoparticles in [9]. It is important to note that both Ag and Al nanoparticle cells suffer from the loss backscattering from metal nanoparticles into the air, as shown in Eq. 4. For the cells with Al nanoparticles, we obtained a better fit to the experimental data by incorporating the Al-Al_2_O_3_ core-shell particle structure into the model, as seen in Fig. 4, in particular, for the case of smaller nanoparticles. Plasmonic effect for the cell with the 60-nm-diameter Al nanoparticles is weak owing to the small *Q*_ext_ and thus *Φ* at far resonance, as shown in Figs. 8 and 9, which show the *Q*_ext_ and *Φ* spectra for the experimental data of cells with Al nanoparticles in [9]. The photocurrent enhancement peak around the GaAs band-edge for the cell with larger, 150-nm-diameter, Al nanoparticles was similar to the case of Ag nanoparticles. Incidentally, the lack of the experimental plots in the shorter-wavelength region for the cell with 150-nm-diameter Al nanoparticles was owing to a temporal error in our spectrometer. Also note that the lack of the model plots above the GaAs band-edge for both the Ag and Al nanoparticle cases is because of no absorption in GaAs, due to the zero imaginary part of the GaAs dielectric constant for the literature parameter [23] in our calculation. Both the 60- and 150-nm-diameter Al nanoparticles reduce cell photocurrent below 800 nm owing to the backscattering into the air and relatively lower *η*_rad_ compared with the case of Ag nanoparticles, as seen in Figs. 5 and 10. The 60-nm-diameter Al nanoparticles yield higher photocurrent for wavelengths in the 500-800-nm range compared with the case of 150-nm-diameter Al nanoparticles, simply owing to low *Φ* for smaller Al nanoparticles, below 0.2 for *λ* > 500 nm for the 60-nm-diameter Al nanoparticles, as seen in Fig. 9. In this near-IR region, Al nanoparticles exert significantly weaker effect on the cells than the Ag nanoparticles, owing to such a small *Φ* (Fig. 9). This follows from the small *Q*_ext_ because the resonant frequency for the Al nanoparticles is ~450 nm, while that for the Ag nanoparticles is in the near-IR range, as found from the calculated *Q*_ext_ shown in Figs. 6 and 8.

**Fig. 11:**
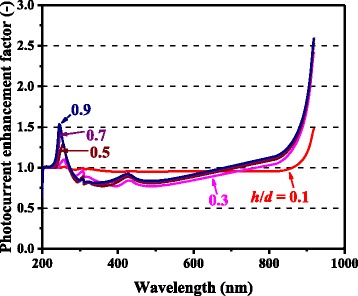
(Color online) Calculated spectral photocurrent enhancement factors for Ag nanoparticles with the diameter of 150 nm and surface coverage fraction of 0.3. The results are shown for different *h/d* ratios

Figure 11 shows the photocurrent enhancement factor spectra for different aspect ratios, *h/d*, of Ag nanoparticles, for *d* = 150 nm and *ξ* = 0.3, calculated by using our model. The photocurrent enhancement curves are nearly identical among relatively high nanoparticles throughout the wavelength range. Very short nanoparticles exhibit little plasmonic effect and the photocurrent enhancement factors are close to unity because of their too small *Q*_ext_. The peaks around 250 nm are incidentally due to the local maxima of the convolution of *η*_rad_ and *Φ*, as understood from Figs. 5 and 7 for instance, where the metal nanoparticles efficiently contribute.

**Fig. 12:**
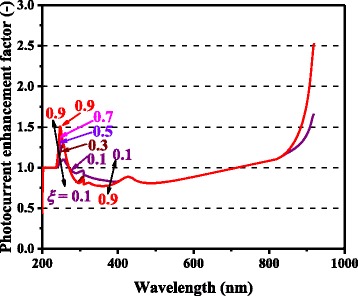
(Color online) Calculated spectral photocurrent enhancement factors for Ag nanoparticles with the diameter of 150 nm and *h/d* ratio of 0.5. The results are shown for different surface coverage fractions

Figure 12 shows the calculated photocurrent enhancement factor spectra for different *ξ* of Ag nanoparticles, for *d* = 150 nm and *h* = 75 nm. The photocurrent enhancement curves are nearly identical throughout the entire wavelength region, except for very small *ξ*. This result follows because *Φ* is close to unity for a wide range of wavelengths, even for relatively small *ξ* for such large nanoparticles, as seen in Fig. 7. Thus, by properly choosing the nanoparticle size, the preparation of highly dense nanoparticle arrays can be avoided.

**Fig. 13:**
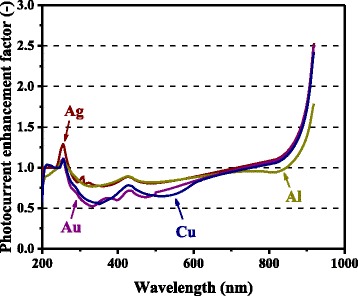
(Color online) Calculated spectral photocurrent enhancement factors for different metal-element nanoparticles with the diameter of 150 nm, height of 75 nm, and surface coverage of 0.3

Figure 13 summarizes the calculated photocurrent enhancement factors for different metals. These calculations were conducted for *d* = 150 nm, *h* = 75 nm, and *ξ* = 0.3. Note that this particle size was chosen as a saturated, sufficiently large size optimized for the photocurrent enhancement, accounting for the series of our calculation results represented by Figs. 3 and 4. The curve for Al is for *t* = 0, which exhibits higher integrated photocurrent enhancement than for the cases with Al_2_O_3_ shells or native oxide layers, shown in Fig. 4. Au and Cu suffer from the absorption loss owing to the surface plasmon resonance in the 300-600-nm range. Above 800 nm, all of the metals exhibit similar spectral photocurrent enhancement factors except for Al, for which the value is somewhat lower. In view of these aspects, Ag may be the most suitable metal for nanoparticle-enhanced photovoltaic devices.

**Fig. 14:**
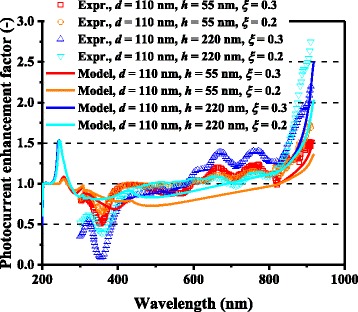
(Color online) Spectral photocurrent enhancement factors of optically thin GaAs solar cells with Ag nanoparticles for the series of data reported in Fig. 3d in [9], including prolate spheroidal particles

So far, we have investigated the photocurrent enhancement in solar cells with oblate spheroidal metal nanoparticles. For prolate spheroidal particles, which we partly used in [9], the formulation for the geometrical factor becomes, instead of Eqs. 13 and 14,

20 $$ {L}_1=\frac{1}{2}\left\{1-\frac{1-{e}^2}{e^2}\left(-1+\frac{1}{2e} \ln \frac{1+e}{1-e}\right)\right\}, $$

21 $$ e=\sqrt{1-\frac{a^2}{c^2}}, $$

where *a* and *c* denote the radii of the minor (horizontal) and major (parallel to the incident light) axes, respectively [13], and thus still *d* = 2*a* and *h* = 2*c*. Figure 14 shows the computed photocurrent enhancement factors along with the experimental data from Fig. 3d in [9], including prolate spheroidal Ag nanoparticles. The model calculation results are in a modest qualitative agreement with the experimental results, while the partial mismatch can be attributed to the experimental particle shape discrepancy from the ideal, regular spheroids, particularly for the tall Ag particles, as seen in the corresponding scanning electron microscope images in [9]. The partial overlap in the model curves for high and low *ξ*s is due to the saturation of *Φ* (=1) even for the low-particle-density conditions.

**Fig. 15:**
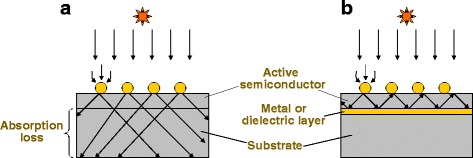
(Color online) Schematic of metal-nanoparticle-enhanced solar cell structures with **a** an absorbing substrate and **b** a back reflector

**Fig. 16:**
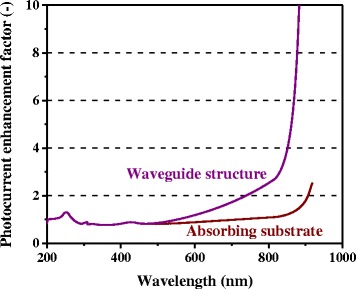
(Color online) Calculated spectral photocurrent enhancement factors for Ag nanoparticles with the diameter of 150 nm, height of 75 nm, and surface coverage of 0.3, with and without a waveguide structure

In our experiments reported in [9], we obtained enhancement in net photocurrent, not only for the longer wavelength range, and enhancement in efficiency for certain conditions. However, in the present cell structures, we observed only slight enhancement in photocurrent and efficiency because of the thick absorbing GaAs substrate. Optical waveguide-like structures with higher refractive indices contrast with a thin photovoltaic layer, and a low-index substrate layer underneath would yield much stronger photocurrent enhancement. Figure 15 shows the schematics of metal-nanoparticle-enhanced solar cell structures with an absorbing substrate and a back reflector. Such a waveguide-type scheme can be realized by inserting a low-index dielectric layer or a metal layer as a mirror-functioning layer at the bottom of the photovoltaic active layer (i.e., semiconductor *p-n* diode layer). In this way, the energy flux direction can be converted from normal to lateral relative to the photovoltaic layer. Figure 16 shows the photocurrent enhancement factor spectra of cells with Ag nanoparticles with *d* = 150 nm, *h* = 75 nm, and *ξ* = 0.3, with and without a back reflector. In this calculation, we assumed a perfect reflector for the back-reflection layer with a 100 % reflectivity and no absorption. Also, for the calculation method, the optical path in the GaAs layer was increased from *L* for the conventional solar cells to 2*L*/*cos*θ, by the perfect reflection by the back mirror layer, rather than *L*/*cos*θ for the above cases without the back layer. Furthermore, we assumed that the optical path length becomes infinite for the angular conditions

22 $$ \theta > \arcsin \left(\frac{n_{\mathrm{air}}}{n_{\mathrm{GaAs}}}\right) $$

out of the angular distribution of the light intensity scattered by subwavelength-sized particles in the quasistatic limit shown in Eq. 2, accounting for the waveguide-mode coupling from the total internal reflection. Such a simple modification of the formalism in our calculation enables to test a completely novel device structure, which demonstrates another capability of our model, namely its flexibility and extensibility. Thus, in Fig. 16, we show a significantly higher photocurrent enhancement by adopting such a waveguide-like photovoltaic layer structure and converting the incident sunlight into waveguide optical modes owing to the scattering induced by the metal nanoparticles, which indicates a great potential for the future development of plasmon-enhanced solar cells. Note also that the presently investigated scheme for utilization of optical waveguide modes differs from another enhancement scheme for utilization of surface plasmon modes [29] by coupling the incident light into surface plasmon polaritons propagating at semiconductor/metal interfaces via some subwavelength-sized features such as nanoscale grooves [30–33].

## Conclusions

In this work, we developed a relatively simple optical model for photocurrent enhancement by plasmonic metal nanoparticles atop solar cells. Our model considers the absorption, reflection, and scattering of the incident sunlight as well as the radiation efficiencies on metallic nanoparticles. Our calculation results satisfactorily reproduce a series of experimental spectral data in [9] for optically thin GaAs solar cells with Ag and Al nanoparticles of various dimensions, demonstrating the validity of our modeling scheme. We fitted our model calculations for the experimental results of GaAs solar cells in this study, but needless to say, our highly generalized model presented in this study is applicable for any kind of photovoltaic material. Our model can be used as a powerful tool for investigations of surface plasmon-enhanced thin-film solar cells to provide design principles for the improvement of device performance.
